# Comparison of Dynamic Magnetic Resonance Defecography With Clinical Examination in Diagnosing Pelvic Floor Dysfunction: An Observational Study

**DOI:** 10.7759/cureus.51378

**Published:** 2023-12-31

**Authors:** Sanajana Wadhwani, Chetana Ratnaparkhi, Avinash Dhok

**Affiliations:** 1 Department of Radiodiagnosis and Imaging, National Cancer Institute, Nagpur, IND; 2 Department of Radiodiagnosis, All India Institute of Medical Sciences, Nagpur, IND

**Keywords:** pelvic organ prolapse, pelvic floor dysfunction, dynamic mri, dynamic magnetic resonance defecography, clinical examination

## Abstract

Background: Pelvic floor dysfunction (PFD) is frequently reported in both sexes. Dynamic magnetic resonance defecography (DMRD) is the preferred modality, mainly due to its superiority and complementary role in clinical examination. However, studies from the perspective of Indian patients are scarce and mostly restricted to females. Thus, we assessed the diagnostic performance of DMRD in patients with PFD and correlated the findings with those on clinical examination.

Materials and methods: This prospective, observational study involved 57 adult patients of either sex, presenting with pelvic floor symptoms (PFS) and diagnosed with PFD. Initially, the patients underwent clinical examination, and diagnosis was recorded. Subsequently, the patients were subjected to DMRD. The findings were correlated with the Pearson "r" correlation coefficient.

Results: A significantly greater proportion of patients had involvement of multiple compartments (36 vs. 12, p<0.001), cystocele (23 vs. 8, p=0.002), and rectal prolapse (25 vs. 14, p=0.030) on DMRD than clinical examination, while there was no significant difference regarding uterine prolapse (p=0.789). Grading of cystocele and rectal prolapse as well as diagnosis of enterocele/peritoneocele, rectocele, and intussusception could be done only with DMRD. DMRD had a strong and significant correlation with clinical examination regarding cystocele (r=0.943, p=0.003), uterine prolapse (r=0.972, p=0.001), and rectal prolapse (r=0.951, p=0.001).

Conclusions: DMRD demonstrated significantly better performance in the diagnosis of multiple compartment involvement, cystocele, and rectal prolapse. DMRD and clinical examination were significantly correlated regarding the diagnosis of cystocele, uterine prolapse, and rectal prolapse. Thus, DMRD provides information, in addition to the clinical examination, and should be used in symptomatic patients.

## Introduction

As a collective term, pelvic floor dysfunction (PFD) is defined as abnormal pelvic floor function and comprises conditions that can have significant adverse impacts on quality of life [1]. PFD is a result of the involvement of all three compartments of the pelvic floor: weakened support of muscles, ligaments, and fasciae [2]. Globally, the prevalence of PFD ranges between 1.9% and 46.50% [3]. Though both sexes are frequently affected, prevalence is higher among females and those with advancing age [4,5].

The pelvic floor, a complex mechanical structure comprising superficial perineal muscles, levator ani, endopelvic fascia, pelvic nerves, and ligaments, presents challenges to surgeons and anatomists due to its intricate and narrow anatomy. It houses visceral components, including intestines, urologic structures, and reproductive organs, and is designed for content suspension and coordinated actions during bladder and rectal emptying. Support for pelvic organs is derived from connections to the pelvis and associated muscles, including the levator ani, anal sphincter complex, pelvic sidewall, and anterior perineal muscles, but disruption in structural and functional interactions may result in multicompartmental dysfunction [6].

PFD can be attributed to varying factors, including trauma, pregnancy, pelvic surgery, or degeneration, and results in a group of pelvic floor symptoms (PFS) that range from lower urinary tract symptoms (LUTS), bowel symptoms, sexual problems, and genito-pelvic pain to pelvic organ prolapse (POP) [7]. Together these symptoms produce serious health concerns, as by the age of 80 years, there is an 11% risk of undergoing an index procedure for prolapse or incontinence, and 17-29% of these patients require a second procedure [8]. The presence of PFS negatively affects the quality of life [3]. Due to improved medical facilities, life expectancy has increased, and PFS burden is expected to rise.

Usually, a multidisciplinary approach is required to reach a diagnosis of PFD, and relying on clinical examination alone for reaching the definitive diagnosis can be challenging, especially in patients with involvement of multiple compartments and/or posterior vaginal wall prolapse [8]. In 45-90% of patients, clinical examination results in either underdiagnosis of the compartments involved or misdiagnosis of the prolapse site, resulting in imprecise treatment choices and subsequent recurrence [8,9]. Additionally, it is not reliable for evaluation of the evacuation anomalies [9]. Pelvic floor weakness generally involves more than one compartment; thus, all the compartments should be examined simultaneously [10].

Over the years, several radiological investigations, including micturating cystourethrography, fluoroscopic defecography, endoanal ultrasound, pelvic magnetic resonance imaging (MRI), endoanal MRI, and others, have come up. Though these investigations are useful in evaluating pelvic pathologies, they are inadequate in assessing the pelvic floor function [11]. On the other hand, magnetic resonance defecography (MRD) is reported to aid in the diagnosis of PFD, especially in the differential diagnosis of POP as well as evacuatory and continence disorders. Dynamic magnetic resonance defecography (DMRD) allows simultaneous assessment of all the pelvic compartments [12]. MRI of the pelvic floor, illustrating the involved organ, was first introduced by Yang et al. and Kruyt et al. in 1991 [13]. The diagnostic performance of DMRD is identical to conventional defecography, and thus, it can be used as an alternative modality [14].

DMRD acts as a critical modality in the diagnosis of POP in complex cases with the involvement of multiple compartments and multiple pelvic organs. Moreover, in complex cases, DMRD permits more effective preoperative planning and selection of a more optimal surgical method [15]. Though the literature from Western countries highlights the utility of DMRD, such studies from the perspective of Indian patients are scarce [11,16-18]. Moreover, studies are restricted to mostly female sex [16,18]. Thus, the present study assessed the diagnostic performance of DMRD in patients with PFD and simultaneously compared the findings with those on clinical examination.

## Materials and methods

This prospective, observational study was performed in the Department of Radiodiagnosis of a tertiary care hospital, over a period of 24 months (November 2018 to October 2020). The study included adult patients aged 18 years or more, of either sex, presenting with PFS, and diagnosed with PFD. While, the patients with pacemakers, metallic implants, a history of claustrophobia, and not willing for DMRD were excluded. The study was carried out by following the principles of the Declaration of Helsinki, 2013. The study began after obtaining approval of the Institutional Ethics Committee and written informed consent of the enrolled patients.

Following enrollment, a detailed clinical history was taken. An experienced urologist for males and urogynecologist for females assessed the POP by performing a clinical examination with the patients in the upright position and straining maximally. Three compartments of the pelvic floor, including anterior (urinary bladder and urethra), middle (uterus with cervix and vagina), and posterior (rectum with anus), were examined [2]. The presence of prolapse of a particular organ on clinical examination was noted, and the size of the prolapse was graded.

Anterior compartment abnormalities mainly included cystocele, defined as the descent of the bladder base below the border of the pubic symphysis. At DMRD, a cystocele was defined as descent of bladder base more than 1 cm below the pubococcygeal line (PCL), and its severity was graded with respect to the position of the bladder base below the PCL: grades 0, 1, 2, and 3 denoting the presence of bladder base <1 cm, 1-3 cm, 3-6 cm, and >6 cm below PCL, respectively [2].

Middle compartment abnormalities included uterine with cervix or vaginal vault prolapse, defined as descent of the vaginal vault or cervix below the PCL. In uterine prolapse, the cervix is located abnormally low through the vagina, which thus appears shortened. In complete uterine prolapse, the vaginal walls are everted, and the uterus is visible as a bulging mass outside the external genitalia. The vaginal vault prolapse was graded as grade 0 suggested by the presence of a vaginal vault above PCL, while grades 1, 2, and 3 represented the presence of vaginal vaults 0-3 cm, 3-6 cm, and >6 cm below PCL, respectively. Additionally, enterocele suggests the herniation of the small intestine into the rectogenital space and may contain fat (peritoneocele) [2].

Posterior compartment abnormalities included rectocele and rectal prolapse. The former is characterized by abnormal bulging of the rectal wall, measured as the depth of wall protrusion beyond the expected margin of the normal rectal wall. It is clinically significant when the bulge exceeds 2 cm during defecation, and usually involves the anterior wall but may rarely involve the posterior wall. The latter involves full-thickness rectal wall prolapse involving both the mucosa and the muscular layer termed prolapse, invagination, or intussusception. The distance of parietal inversion from the anal verge was assessed while evaluating invagination and further classified as intra-rectal (distal, middle, or proximal with respect to rectal length), intra-anal, or extra-anal. Extra-anal invagination is termed as rectal prolapse. Low- and high-grade intussusceptions were defined as in-folding of rectal mucosa but not entering the anal canal and a prolapse that penetrates the anal canal or impedes evacuation, respectively [2].

DMRD examination

Four hours before the scan, a rectal cleansing enema was given to the patients. Additionally, two hours prior, patients were asked to micturate so that the bladder was moderately filled. The patients were explained the procedure so as to get maximum compliance and offered protective pads or diaper pants to make them comfortable during the scan.

**Figure 1:**
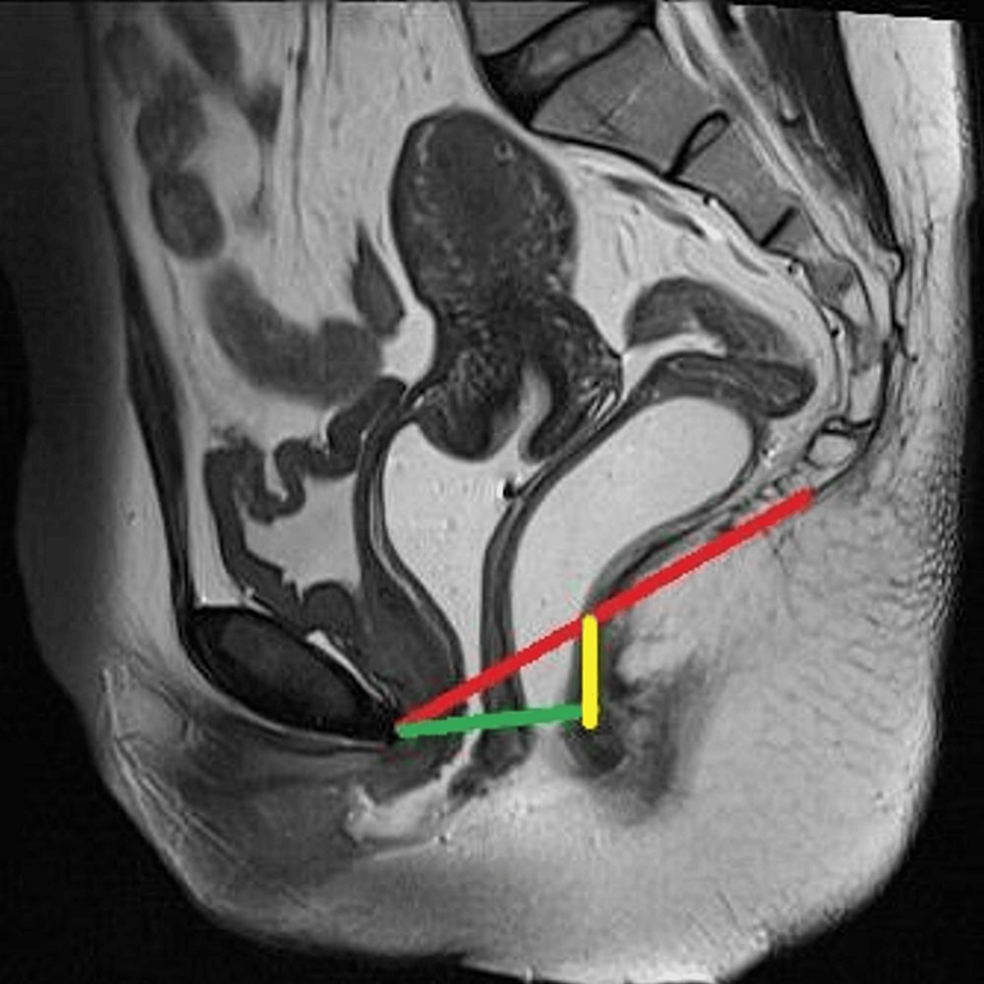
T2 weighted image (mid-sagittal plane) illustrating reference lines used to calculate pelvic organ descent on DMRD scans: H line (green), M line (yellow), and pubococcygeal line (red)

The patients were taken to the MRI room, positioned in a lateral decubitus position, and jelly was instilled into the rectum and then in the lithotomy position for instilling jelly into the vagina. The examination was performed with a 1.5 Tesla MRI machine (16 channel GE 1.5 Tesla HDXT, Version 23.0) with the phased array coil. To ensure complete visualization of prolapsed organs, the coil was centered low on the pelvis, and the patient was instructed to squeeze as if trying to prevent the leaking of urine or feces and hold this position for the duration of the sequence. For maximum straining, the patients were instructed to bear down as much as possible as if they were constipated and trying to defecate. For the evacuation phase, the patients were instructed to repeat the evacuation process until the rectum was emptied. Table 1 depicts the protocol for DMRD evaluation of PFD. Figure 1 illustrates reference lines used to calculate pelvic organ descent on DMRD scans.

**Table 1:** Protocol for DMRD evaluation of PFD *If defecation of rectal gel is difficult then this sequence is repeated over a prolonged interval of 80-120 seconds. TR, repetition time; TE, echo time; FOV, field of view; PFD, pelvic floor dysfunction; DMRD, dynamic magnetic resonance defecography

Pulse sequence	Imaging plane	TR/TE (msec)	FOV (cm)	Slice thickness (mm)	Study phase
T1 weighted localizer	-	-	Large	-	Rest
T2 weighted fast spin echo	Axial, coronal, sagittal	4800/100	25×25	5	Rest
FIESTA	Mid-sagittal	4.8/2.2	40×40	6	Straining
FIESTA	Mid-sagittal	4.8/2.2	40×40	6	Squeezing
FIESTA*	Mid-sagittal	4.8/2.2	40×40	6	Defecation

The sample size was determined considering uterine parameters as the main outcome. The following assumptions were made from the study by El Sayed et al. [19]. With a power of 80%, alpha error of 5%, mean anterior cervical lip position of 4.6 cm, and standard deviation of 0.78 cm, the required sample size was found to be 57.

Statistical analyses

The data was analyzed with SPSS (IBM, Armonk, NY, USA) version 23.0 for Windows. The categorical and continuous variables are represented as frequency (percentage) and mean (standard deviation, SD), respectively. The chi-square test was used to assess the association between categorical variables. The Pearson "r" correlation coefficient was used to find the correlation between clinical examination and MRI scan. A two-tailed probability value of <0.05 was considered statistically significant.

## Results

Table 2 depicts the baseline characteristics of the enrolled patients. The mean age of the study population was 48.72 (14.63) years, and patients mainly comprised of female sex (75.4%). Among risk factors, a history of strenuous work, asthma, and COPD were present in 52.6%, 14.1%, and 3.5%, respectively. Additionally, 46.51% and 48.84% of patients had 1-2 and >2 vaginal deliveries, respectively. Assessment of PFS revealed that complaints of the urinary system, incomplete defecation, mass coming out of the anus, and something coming out of the vagina were observed in 28.1%, 28.1%, 10.5%, and 88.37% of patients, respectively.

**Table 2:** Baseline characteristics

Characteristics	N (=57)	%
Age, years, mean (SD)	48.72 (14.63)	
Sex, n (%)		
Male	14	24.6
Female	43	75.4
Strenuous work, n (%)	30	52.6
Asthma, n (%)	8	14.1
COPD, n (%)	2	3.5
Number of vaginal deliveries, n=43, n (%)		
0	2	4.65
1-2	20	46.51
> 2	21	48.84
Symptoms, n (%)		
Urinary complaints	16	28.1
Incomplete defecation	16	28.1
Mass coming out of anus	6	10.5
Something coming out of vagina, n=43	38	88.37

Table 3 depicts the comparison of findings on clinical and DMRD examinations. On clinical examination, nine (15.79%) patients had involvement of no compartments, while a significantly greater proportion of patients had involvement of multiple compartments on DMRD relative to clinical examination (36 vs. 12, p<0.001). On DMRD, cystocele was observed in a significantly greater proportion of patients relative to clinical examination (23 vs. 8, p=0.002). Of 23 patients with cystocele observed on DMRD, 17 had grade 1 and six had grade 2 cystocele. Similarly, examination with DMRD resulted in a significantly greater proportion of patients with rectal prolapse relative to clinical examination (25 vs. 14, p=0.030). On DMRD, among 25 patients with rectal prolapse, 16 and nine patients had mild and moderate to severe rectal prolapse, respectively. However, the findings on clinical and DMRD examination did not differ significantly regarding uterine prolapse (p=0.789). Additionally, though not observed on clinical examination, DMRD led to the diagnosis of enterocele/peritoneocele, rectocele, and intussusception in 26, 20, and three patients, respectively. Of 20 patients with rectocele, 16 had anterior and four had posterior rectocele.

**Table 3:** Comparison of clinical and DMRD findings DMRD, dynamic magnetic resonance defecography

Characteristics	Clinical findings	DMRD findings	P
Compartments involved, n (%)			<0.001
None	9 (15.79)	0 (0)	
Single	36 (63.16)	21 (36.84)	
Multiple	12 (21.05)	36 (63.16)	
Cystocele, n (%)	8 (14.04)	23 (40.35)	0.002
Grade 1	-	17 (29.82)	
Grade 2	-	6 (10.53)	
Uterine prolapse, n=43, n (%)			0.789
Grade 1	12 (27.91)	10 (23.26)	
Grade 2	15 (34.88)	14 (32.56)	
Grade 3	16 (37.21)	19 (44.19)	
Rectal prolapse, n (%)	14 (24.56)	25 (43.86)	0.030
Mild	-	16 (28.07)	
Moderate to severe	-	9 (15.79)	
Enterocele/peritoneocele, n (%)	-	26 (45.61)	-
Intussusception, n (%)	-	3 (5.26)	-
Rectocele, n (%)			
Anterior	-	16 (28.07)	-
Posterior	-	4 (7.02)	-

**Figure 2:**
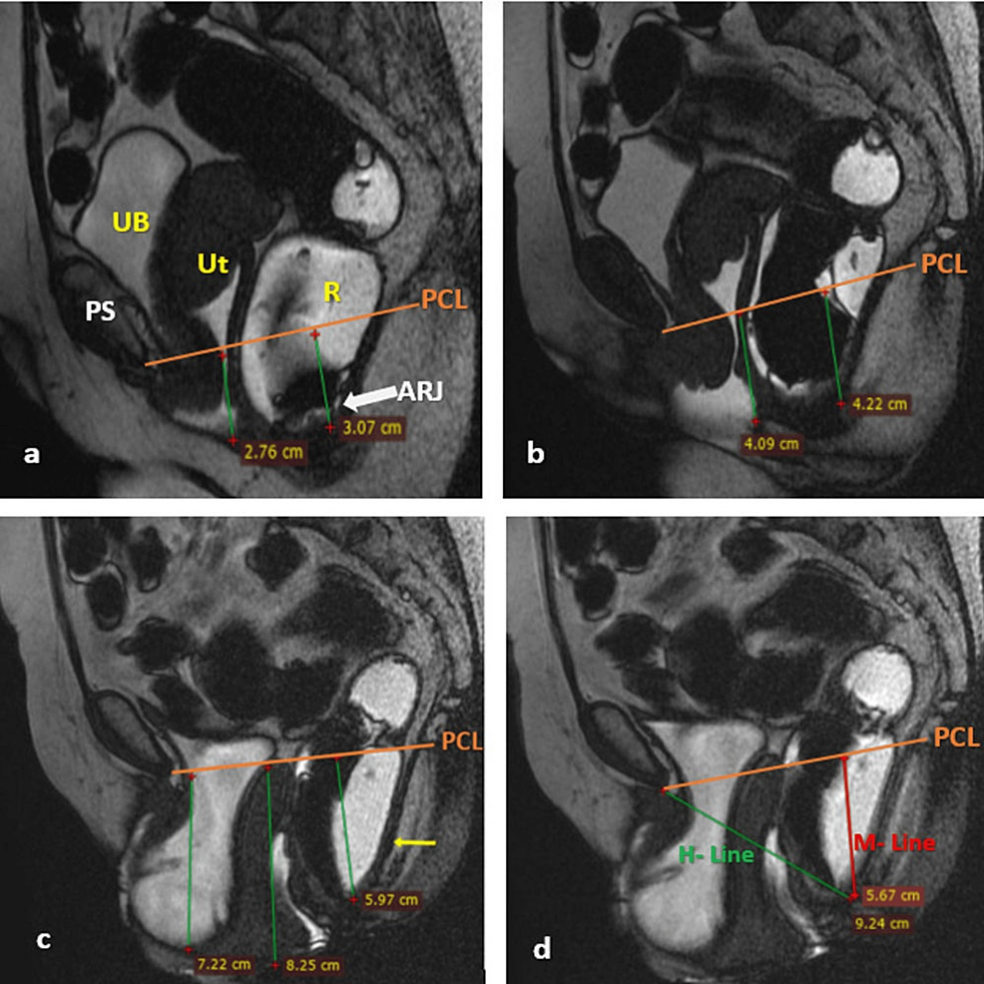
DMRD scan (mid-sagittal plane) illustrating grade 2 cystocele with other compartment abnormalities At rest (a): lower uterine segment prolapse grade 1 (green line) and mild rectal prolapse (green line), while UB is above the PCL (orange line); straining phase (b): lower uterine segment prolapse grade 2 (green line) and moderate rectal prolapse (green line), while UB is above the PCL (orange line) during maximum straining; defecation phase (c): significant finding of cystocele (green line), uterine prolapse grade 3 (green line), and moderate to severe rectal prolapse (green line) with caudal angulation of the levator plate (yellow arrow); and (d): moderate hiatal enlargement depicted by H line (green line) and moderate to severe rectal prolapse depicted by M line (red line). Thus, the patient had involvement of all three compartments. UB, urinary bladder; Ut, uterus; R, rectum; PS, pubic symphysis; PCL, pubococcygeal line; ARJ, anorectal junction; DMRD, dynamic magnetic resonance defecography

Figure 2 illustrates the DMRD scan of a patient with cystocele with the involvement of other compartments.

**Figure 3:**
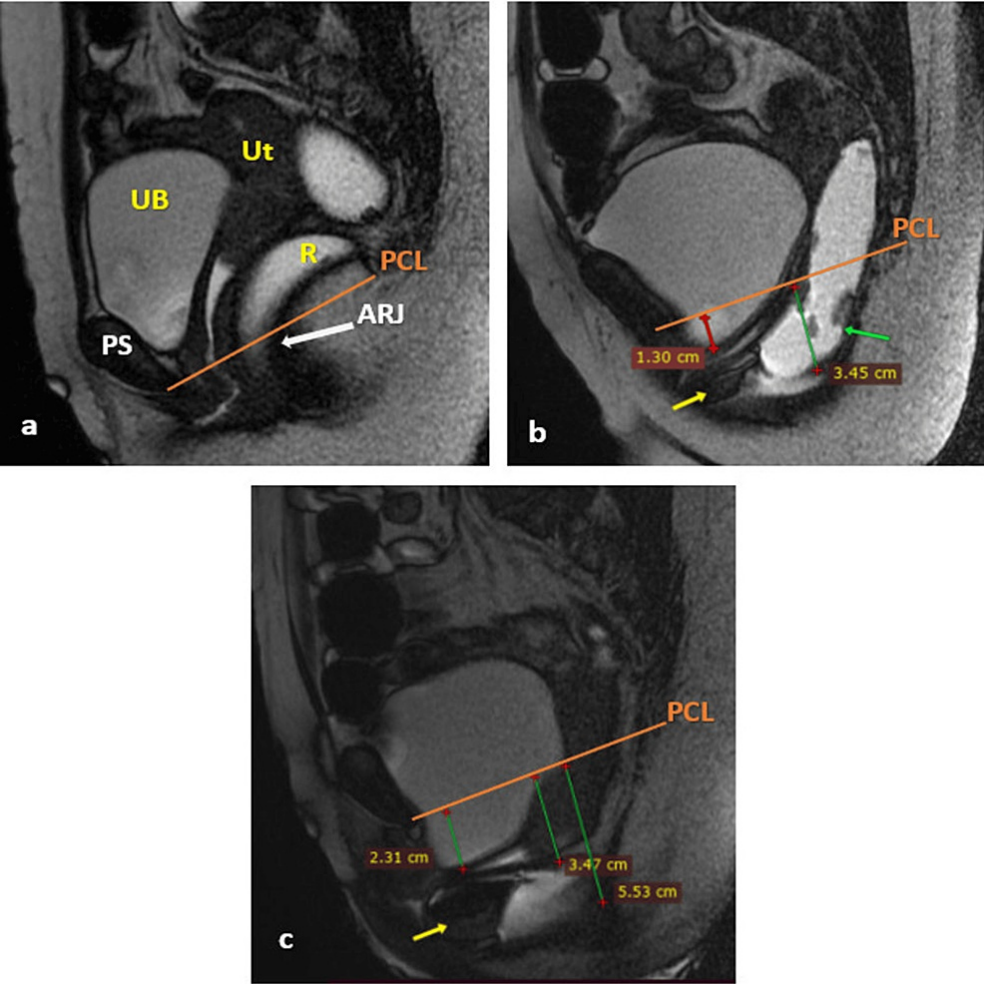
DMRD scan (mid-sagittal plane) illustrating uterine prolapse with other compartment abnormalities At rest (a): no obvious pathology other than elongated cervix; straining phase (b): mild bladder descent (red line), moderate rectal prolapse (green line), associated intussusception (green arrow), and anterior rectocele (yellow arrow); and defecation phase (c): mild cystocele (green line), lower uterine prolapse grade 2 (green line), moderate to severe rectal prolapse (green line), and anterior rectocele (yellow arrow). Thus, the patient had pathology in all three compartments. UB, urinary bladder; Ut, uterus; R, rectum; PS, pubic symphysis; PCL, pubococcygeal line; ARJ, anorectal junction; DMRD, dynamic magnetic resonance defecography

Figure 3 illustrates the DMRD scan of a patient with uterine prolapse with the involvement of other compartments.

**Figure 4:**
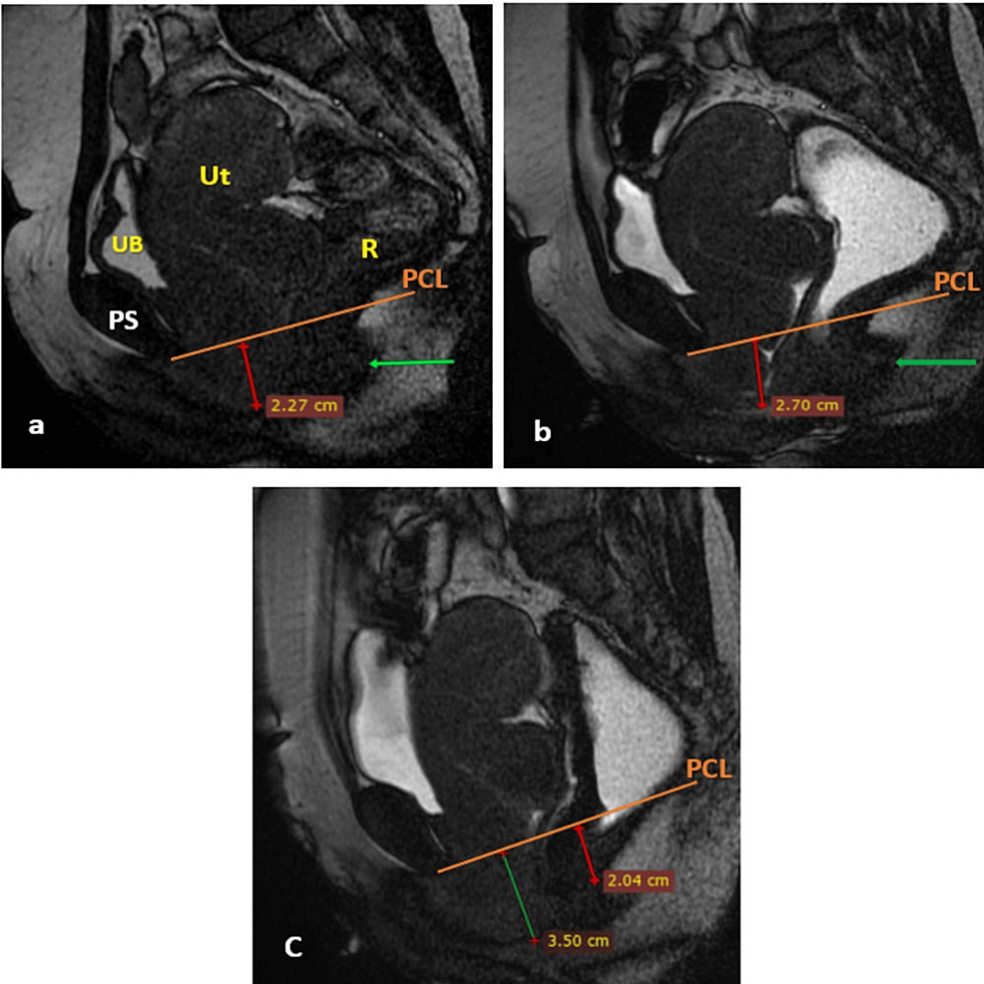
DMRD scan (mid-sagittal plane) illustrating rectocele with middle compartment abnormality At rest (a): lower uterine segment prolapse grade 1 (red line) and mild posterior rectocele (green arrow) with UB located above the PCL (orange line); straining phase (b): lower uterine segment prolapse grade 1 (red line) and mild posterior rectocele (green arrow) with UB located above the PCL during maximum straining; and defecation phase (c): lower uterine segment prolapse grade 2 (green line) and mild rectal prolapse (green line) with UB located above the PCL. Thus, the patient had pathology of middle and posterior compartments, observed on rest and confirmed on DMRD with a change in the grade on DMRD. UB, urinary bladder; Ut, uterus; R, rectum; PS, pubic symphysis; PCL, pubococcygeal line; ARJ, anorectal junction; DMRD, dynamic magnetic resonance defecography

Figure 4 illustrates the DMRD scan of a patient with a rectocele and involvement of the middle compartment.

Table 4 depicts the correlation of findings on DMRD with clinical examination. The findings on DMRD examination had a positive, strong, and statistically significant correlation with clinical examination regarding cystocele (r=0.943, p=0.003), uterine prolapse (r=0.972, p=0.001), and rectal prolapse (r=0.951, p=0.001).

**Table 4:** Correlation of findings on DMRD with clinical examination

DMRD versus clinical	r	p
Cystocele	0.943	0.003
Uterine prolapse	0.972	0.001
Rectal prolapse	0.951	0.001

## Discussion

The principal findings of the present study showed significantly better performance of DMRD in the diagnosis of multiple compartments involvement, cystocele, and rectal prolapse than the clinical examination. Additionally, both DMRD and clinical examination had a positive, strong, and statistically significant correlation for the diagnosis of cystocele, uterine prolapse, and rectal prolapse.

Generally, clinical examination is inadequate to evaluate the entire pelvic organs and pelvic floor-related abnormalities. With clinical examination alone, it is difficult to differentiate between cystocele, enterocele, and high rectocele [20]. In a single examination, DMRD permits multiparametric and multiplanar assessment of the soft tissues and organs of the pelvic region, provides both anatomical and functional information, does not use ionizing radiation as well as there is no requirement for patient preparation [21]. Additionally, prolapse in asymptomatic compartments that are occult on physical examination is readily illustrated on DMRD [22]. Available studies report the comparison of findings on DMRD and clinical examination in patients with POP [23-27].

In the present study, there was a strong correlation between DMRD and clinical examinations regarding cystocele, uterine prolapse, and rectal prolapse. A study reported a good correlation between MRD and clinical examination for the diagnosis of cystoceles, rectoceles, enteroceles, or peritoneoceles [28]. Other studies demonstrated a greater correlation of MRD with clinical grading for anterior compartment prolapse relative to middle and posterior compartment prolapse [24-26]. The absence of significant correlation in the middle and posterior compartments highlights the fact that MRD illustrates further anatomic information about these compartments [26]. A relatively recent systematic review and meta-analysis reported no significant difference in the findings on MRD and clinical examination regarding cystocele, rectocele, rectal prolapse, and intussusception; however, MRD resulted in a significantly greater proportion of patients with enterocele [29].

In the present study, a significantly greater proportion of patients were diagnosed with cystocele and rectal prolapse on DMRD than clinical examination. However, grading of both cystocele and rectal prolapse was possible only on DMRD. Usually, enteroceles are not diagnosed on clinical examination. As per the available studies, 13.33-20% of enterocele are diagnosed with MRD in patients without a prior clinical diagnosis of enterocele [30-32]. Another study demonstrated that clinical examination led to the diagnosis of only 30% of total MRD-diagnosed enteroceles and misdiagnosed 10% of these patients as rectoceles [24]. These findings suggest that DMRD is superior to clinical examination for the diagnosis of POP.

In the present study, uterine prolapse could be graded on DMRD and clinical examination, and both diagnostic modalities did not differ significantly. Additionally, the diagnosis of enterocele/peritoneocele (45.61%), rectocele (35.09%), and intussusception (5.26%) could be established only on DMRD. On clinical examination, the underdiagnosis of PFD may be attributed to clinician and/or patient-related factors. The former may involve factors, including obesity, reluctance, or embarrassment associated with symptoms, and poor Valsalva performance, while the latter includes factors such as variation in measurement techniques and examination methods as well as reader variability.

PFD is often multicompartmental, and examination with DMRD revealed that a significantly greater proportion of patients had involvement of multiple compartments relative to clinical examination. This suggests that DMRD leads to findings in addition to clinical examination, particularly in patients with multicompartment involvement. Additionally, MRI is a useful extension of the clinical examination and is more accurate than clinical examination alone in diagnosing POP [20]. Overall, DMRD led to the diagnosis of more pelvic floor anomalies than clinical examination.

The present study had certain limitations. First, a relatively small number of patients were enrolled. Second, x-ray defecography, a gold standard investigation, was not utilized, and thus, findings were not compared. Third, as patients were not followed up, the effect of DMRD findings on treatment plans and outcomes could not be commented upon. Fourth, on clinical examination, a significantly smaller number of patients had multicompartment involvement, and pathologies such as cystocele and rectal prolapse could not be graded, thus clinician and/or patient-related factors were another limitation.

## Conclusions

To conclude, DMRD demonstrated significantly better performance in the diagnosis of multiple compartment involvement, cystocele, and rectal prolapse. Additionally, both DMRD and clinical examination were significantly correlated in the diagnosis of cystocele, uterine prolapse, and rectal prolapse. In patients with PFD symptoms, DMRD provides information, in addition to the clinical examination, and thus, should be used in symptomatic patients, if there are negative findings on clinical examination or involvement of multicompartment is anticipated.
